# Inflammatory thresholds and the species-specific effects of colonising bacteria in stable chronic obstructive pulmonary disease

**DOI:** 10.1186/s12931-014-0114-1

**Published:** 2014-09-14

**Authors:** Richa Singh, Alexander J Mackay, Anant RC Patel, Davinder S Garcha, Beverly S Kowlessar, Simon E Brill, Louise E Donnelly, Peter J Barnes, Gavin C Donaldson, Jadwiga A Wedzicha

**Affiliations:** Airway Disease Section, National Heart and Lung Institute, Imperial College, Dovehouse Street, London, SW3 6LR UK; Centre for Respiratory Medicine, University College London, Royal Free Campus, Rowland Hill Street, London, NW3 2PF UK

**Keywords:** COPD, Inflammation, Bacteria, Colonisation

## Abstract

**Background:**

There has been increasing interest in the use of newer, culture-independent techniques to study the airway microbiome of COPD patients. We investigated the relationships between the three common potentially pathogenic microorganisms (PPMs) *Haemophilus influenzae, Streptococcus pneumoniae* and *Moraxella catarrhalis*, as detected by quantitative PCR (qPCR), and inflammation and health status in stable patients in the London COPD cohort.

**Methods:**

We prospectively collected sputum, serum and plasma samples for analysis of airway bacterial presence and load, and airway and systemic inflammation from 99 stable COPD patients between January 2011 and October 2012. Health status was measured with St George’s Respiratory Questionnaire and COPD Assessment Test.

**Results:**

Airway inflammation and plasma fibrinogen, but not C-reactive protein, were greater in samples with PPM detection (p < 0.001, p = 0.049 and p = 0.261, respectively). Increasing total bacterial load was associated with increasing airway (p < 0.01) but not systemic inflammation (p > 0.05). Samples with high total bacterial loads had significantly higher airway inflammation than both samples without PPM detection and those with lower loads. *Haemophilus influenzae* presence was associated with significantly higher levels of airway but not systemic inflammation for all given pathogen loads (p < 0.05), and was significantly greater than with other PPMs. No association was observed between inflammation and health status (p > 0.05).

**Conclusions:**

Airway and systemic inflammation, as measured by fibrinogen, is greater in stable COPD patients with PPMs detected using the culture-independent qPCR technique. The airway, but not systemic inflammatory response, appears to have a total pathogen-load threshold and appears attributable to *Haemophilus influenzae*. However, discordance between inflammation and health status was observed.

**Electronic supplementary material:**

The online version of this article (doi:10.1186/s12931-014-0114-1) contains supplementary material, which is available to authorized users.

## Background

Chronic obstructive pulmonary disease (COPD) is characterised by airflow limitation and is associated with persistent airway and systemic inflammation, which increases during episodes of acute deterioration, known as exacerbations [1-3]. COPD is currently the third leading cause of death worldwide [4] and exacerbations contribute to the substantial morbidity and mortality, and the considerable health-economic burden associated with this disease [5].

Historically, the tracheobronchial tree and lung parenchyma in healthy, non-smoking individuals were described as sterile, using traditional, culture-based techniques. However, in COPD patients, potentially pathogenic microorganisms (PPMs) are frequently isolated from both sputum and bronchoscopic samples during periods of stability, termed lower airway bacterial colonisation (LABC) [6,7]. *Haemophilus influenzae* (HI) is often the most commonly isolated PPM at stable and exacerbation states, with *Streptococcus pneumoniae* (SP), and *Moraxella catarrhalis* (MC) also frequently isolated [8,9]. LABC has a detrimental effect on the natural history of COPD, with increased airway and systemic inflammation [7,10-12], increased exacerbation frequency [6], and an accelerated lung function decline [13].

Previous LABC studies have used traditional, culture-based microbiological identification. However, as only 1% of bacteria can be cultured using traditional methods [14], there has been increasing interest in the use of culture-independent diagnostic techniques. Many of these rely on the analysis of the conserved bacterial 16S-rRNA gene. Studies using such techniques have demonstrated the presence of a wide range of bacterial species in healthy individuals, described as the core microbiome, which may become disrupted in disease states [15]. However, these techniques are expensive and time-consuming to perform and analyse, limiting their use to small sample numbers. The culture-independent quantitative polymerase chain reaction (qPCR) technique targeting commonly isolated PPMs in COPD; HI, SP, and MC, has been shown to be more discriminatory than culture, detecting a higher prevalence of PPMs at both stable and exacerbation states [16]. This technique is relatively inexpensive and can be used to examine much larger sample numbers.

We hypothesised that using this qPCR technique to accurately detect PPMs, airway and systemic inflammation would be related to both their presence and loads, and that a bacterial load threshold for increased inflammation would be observed, leading to worse health status. Furthermore, we hypothesised that as HI tends to persist in the airways, the higher inflammatory response seen in colonised patients would be attributable to HI rather than the other PPMs.

## Methods

### Patient recruitment

Ninety-nine stable COPD patients enrolled in the London COPD cohort between January 2011 and October 2012 were included. The patients form part of a rolling cohort used to prospectively investigate the mechanisms and aetiology of COPD exacerbations [17]. Patients were included if the post-bronchodilator forced expiratory volume in one second (FEV_1_) was ≤80% and FEV_1_/forced vital capacity (FVC) was <0.7. As per GOLD guidelines, bronchodilator reversibility testing was not required for the diagnosis and assessment of severity [3]. Patients with a history of asthma, primary bronchiectasis or any other significant respiratory diseases were excluded, as were those unable to complete daily symptom diary cards.

### Clinical assessment

At annual review or recruitment, a full medical and smoking history was taken, clinical examination performed and the SGRQ [18] completed. FEV_1_ and FVC were measured in accordance with ATS/ERS guidelines using a Vitalograph Gold Standard spirometer (Vitalograph Ltd, Maids Moreton, UK). Body mass index (BMI) was calculated from height and weight.

Patients completed daily symptom diary cards and were prospectively reviewed in clinic every three months when stable. Stable state was defined as those patients without evidence of symptom-defined exacerbations in the preceding 4 weeks and the subsequent 2 weeks post-clinic visit. At all study visits, patients were asked to complete the CAT. Serum C-reactive protein (CRP) quantification was performed using Modular Analytics E 170 Module (Roche, Burgess Hill, UK) and plasma fibrinogen measured using the Clauss method (IL ACL Top Coagulation Analyzer, Lexington, MA, USA).

### Sputum collection and processing

Patients were asked to spontaneously expectorate sputum samples into a sterile pot. Patients unable to spontaneously expectorate sputum underwent sputum induction [19]. Sputum samples were graded using the BronkoTest® colour chart and processed as soon as possible following collection.

Sputum plugs were separated from contaminating saliva by macroscopic examination using sterile forceps. The sputum was homogenized with standard isotonic phosphate-buffered saline (PBS) with glass beads as previously published [2,20]. A proportion of this preparation was frozen at -80°C and used for later detection of PPMs by qPCR; the remainder was centrifuged and aliquots of supernatant stored at -80°C for subsequent analysis of airway cytokines.

### Routine microbiological culture

Where there was sufficient sputum quantity, one-third was taken for subsequent routine microbiological culture carried out in the Department of Medical Microbiology, Royal Free Hampstead NHS Trust, London, as previously described [16].

### DNA extraction and multiplex qPCR detection of bacteria

qPCR was carried out in the Centre for Clinical Microbiology, University College London, as previously described [16]. Homogenized sputum samples were thawed and processed using a heat-kill treatment at 90°C for 30 minutes before being centrifuged at 13 000 g for 10 minutes. The cell pellet was washed in 1 ml PBS and spun at 13 000 g for another 10 minutes before removal of the supernatant and re-suspension of the pellet in 200 μl of PCR-grade UV-sterilized water (Sigma-W4502). 200 μl of 10% Chelex 100 (Sigma C-7901) was added to each sample and incubated for 20 minutes in a heat block at 56°C. Samples were heated at 95°C for five minutes prior to cooling on ice and subsequently the samples were spun in a microfuge at 16 000 g for 10 minutes. Supernatant containing extracted DNA was transferred to a fresh UV-sterilized 1.5 ml eppendorf tube and stored at 4°C.

Real-time multiplex qPCR was performed on the extracted DNA for SP, HI and MC as previously described [16]. The minimum limit of detection used in this study was 10^4^ colony-forming units (cfu).ml^-1^. Samples were classified to have lower airway bacteria colonisation (LABC) if the qPCR was positive for at least one of these three PPMs.

### Measurement of sputum inflammatory markers

Levels of CXC-chemokine ligand 8 (CXCL8), interleukin (IL)-1β, and myeloperoxidase (MPO) in the sputum supernatants were measured using high sensitivity enzyme-linked immunosorbent assay kits (R&D Systems, Abingdon, UK). The lower limit of detection were 3.5 pg.ml^-1^, <1.0 pg.ml^-1^ and 0.014 ng.ml^-1^ for CXCL8, IL-1β, and MPO respectively.

### Statistical analysis

Data were analysed using GraphPad PRISM version 6.0 (GraphPad Software Inc., San Diego, CA, USA) and PASW Statistics version 21 (SPSS Inc., Chicago, IL, USA). Normally distributed data were expressed as mean and standard deviation (SD) and non-parametric data as median and interquartile range (IQR). Differences between groups were analysed by independent t-test, Mann-Whitney U Test, paired t-test, Wilcoxon-matched pairs, one-way ANOVA or Kruskal-Wallis analysis with multiple comparisons, depending on the sample population being investigated. Sputum cytokines and bacterial load by qPCR were correlated using Spearman’s rank correlation (two-tailed) in a univariate analysis. Relationship between PPM load, PPM species and sputum cytokines were analyzed by multiple regression. Relationship between sputum cytokines, exacerbation frequency, FEV_1_ %predicted and SGRQ or CAT score were analyzed by linear regression in a multivariate analysis. Categorical binary variables were analyzed by χ^2^-analysis. A probability of p < 0.05 was considered to be statistically significant.

Sample colonisation status was considered as individual events when describing the relationship to inflammation, as the associated PPMs (HI, SP or MC) could differ within the same individual and only cross-sectional analyses were performed. When colonisation status was related to patient characteristics, only the first sample was used from each patient to avoid complications with repeated measures. SGRQ and CAT scores were analyzed with inflammatory markers taken on the same day.

### Ethical considerations

The study was approved by the Royal Free Hospital Research Ethics Committee (09/H0270/8). All patients gave written informed consent.

## Results

### Patient characteristics

Ninety-nine COPD patients provided 183 sputum samples for analysis. Their baseline characteristics are reported in Table 1. Patients were sub-grouped into colonised (LABC) and non-LABC based on their sputum sample at study recruitment. There were no significant differences in baseline characteristics between the two groups.

**Table 1:** Clinical characteristics of stable COPD patients, and by colonisation status* at study onset

	**Overall**	**Non-colonised**	**Colonised**	**p-value** ^**†**^
	**n = 99**	**n = 64**	**n = 35**	
	Mean (SD)	Mean (SD)	Mean (SD)	
**Age (years)**	72.1 (8.9)	72.0 (9.3)	72.0 (9.3)	0.947
**FEV**_**1**_ **(L)**	1.32 (0.54)	1.36 (0.59)	1.24 (0.44)	0.318
**FEV**_**1**_ **(% predicted)**	51.5 (21.6)	53.3 (21.7)	48.1 (21.4)	0.257
**FVC (L)**	2.80 (0.86)	2.84 (0.93)	2.74 (0.73)	0.607
**FEV**_**1**_ **/ FVC ratio (%)**	47.2 (13.0)	48.0 (13.4)	45.9 (12.3)	0.440
**BMI (kg/m** ^**2**^ **)**	26.6 (5.8)	26.9 (5.8)	26.2 (5.8)	0.591
	Median (IQR)	Median (IQR)	Median (IQR)	
**Exacerbation frequency**	2.00 (1.00-3.00)	1.97 (1.04-3.03)	2.04 (1.00-2.89)	0.511
**Smoking pack years**	48.4 (24.4-67.5)	47.2 (25.4-64.7)	51.0 (25.4-105.6)	0.213
**ICS dose (beclomethasone equivalent μg)**	1000 (500-2000)	1000 (500-2000)	1000 (500-2000)	0.224
	N (%)	N (%)	N (%)	
**Male gender**	66 (67)	40 (63)	26 (74)	0.234
**Current Smokers**	34 (34)	23 (36)	11 (31)	0.651
**Chronic Bronchitis**	78 (79)	49 (77)	29 (83)	0.464

*Bronchial colonisation defined as sputum positive for *Haemophilus influenzae* (HI)*, Streptococcus pneumoniae* (SP) and/or *Moraxella catarrhalis* (MC) using quantitative polymerase chain reaction (qPCR).

Definitions: ICS = inhaled corticosteroids; BMI = body mass index.

^**†**^p-value refers to unpaired t-test between non-colonised and colonised patients.

### Sputum bacterial isolates and loads

One or more PPMs were identified by qPCR in 64/183 (35%) of sputum samples and these samples were defined as LABC. Single HI or SP isolation was equally prevalent, each identified in 21/64 (33%) of positive samples. Mixed-PPM detection (>1 PPM) was identified in 15/64 (23%) of samples, and single MC detected in 7/64 (11%).

The mean bacterial load for all PPMs was 10^7.1(SD1.7)^ cfu.ml^-1^. The bacterial loads detected for individual HI, SP, MC, and mixed-PPM isolates were significantly different (10^6.2(1.2)^ vs. 10^6.5(1.1)^ vs. 10^8.6(1.8)^ and 10^8.5(1.5)^ cfu.ml^-1^ respectively, p < 0.001). When each species load was compared, HI and SP loads were similar to each other (p > 0.05) but were both significantly lower than MC and mixed-PPM loads (all, p < 0.001).

Qualitative bacterial culture, in addition to qPCR data, was available in 116/183 (63%) samples where sufficient sputum was obtained. Of these, 17/116 (11%) samples had PPMs identified on culture; 7 HI, 1 MC, 5 SP, 2 *Pseudomonas aeruginosa*, 1 *Staphylococcus aureus* and 1 *Proteus vulgaris*, compared to 45/116 (39%) samples which had PPMs identified by qPCR (χ^2^, p < 0.001).

### Bacterial colonisation status and BronkoTest® colour

**Figure 1:**
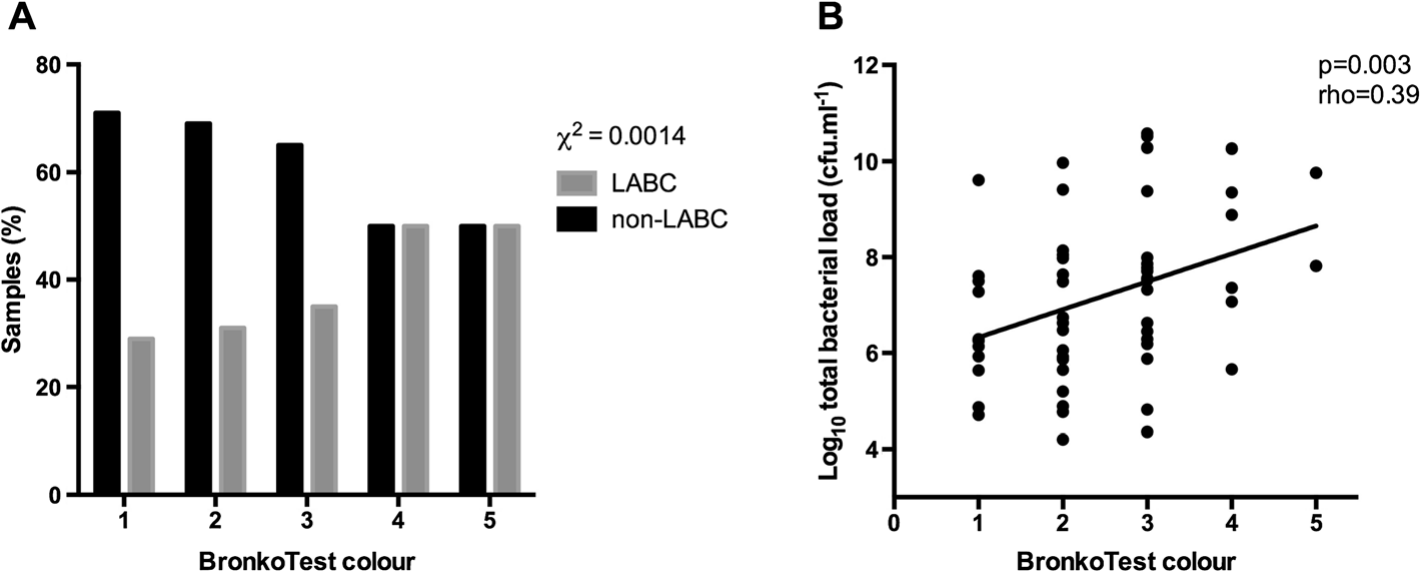
**Bacterial colonisation status and BronkoTest**® **colour. (A)** Proportion of colonised (LABC) and non-LABC sputum samples according to BronkoTest® colour chart. **(B)** Relationship between BronkoTest® colour and total bacterial load by qPCR.

172/183 (94%) sputum samples had colour recorded using the standardised BronkoTest® chart. The proportion of LABC samples for any PPM identified by qPCR was significantly higher with darker (higher BronkoTest® number) sputum (χ^2^, p = 0.001, Figure 1A). In 57/64 (89%) LABC samples where colour was recorded, increasing total bacterial load was significantly associated with higher BronkoTest® colour (rho = 0.39; p = 0.003, Figure 1B), but no significant difference was seen between BronkoTest® colour and the isolation of the different PPMs (p = 0.817).

### Bacterial colonisation and inflammation

LABC samples had significantly higher levels of sputum CXCL8, IL-1β and MPO than non-LABC samples (p < 0.001, Table 2).

**Table 2:** Airway and systemic inflammation in colonised* and non-colonised samples

	**Non-colonised samples**	**Colonised samples**	**p-value**
	**n = 119**	**n = 64**	
**Median (IQR) CXCL8 (ng.ml** ^**-1**^ **)**	83 (24-182)	162 (71-309)	<0.001
**Median (IQR) IL-1β (ng.ml** ^**-1**^ **)**	0.6 (0.2-1.9)	1.4 (0.7-5.9)	<0.001
**Median (IQR) MPO (μg.ml** ^**-1**^ **)**	17.1 (7.6-30.6)	29.5 (16.2-40.9)	<0.001
**Median (IQR) CRP (mg.l** ^**-1**^ **)**	2.0 (1.0-8.0)	2.0 (1.0-5.0)	0.261
**Median (IQR) Fibrinogen (g.l** ^**-1**^ **)**	3.5 (3.2-4.1)	3.8 (3.3-4.4)	0.049

*Bronchial colonisation defined as sputum sample positive *Haemophilus influenzae* (HI)*, Streptococcus pneumoniae* (SP) and/or *Moraxella catarrhalis* (MC) using quantitative polymerase chain reaction (qPCR).

**Figure 2:**
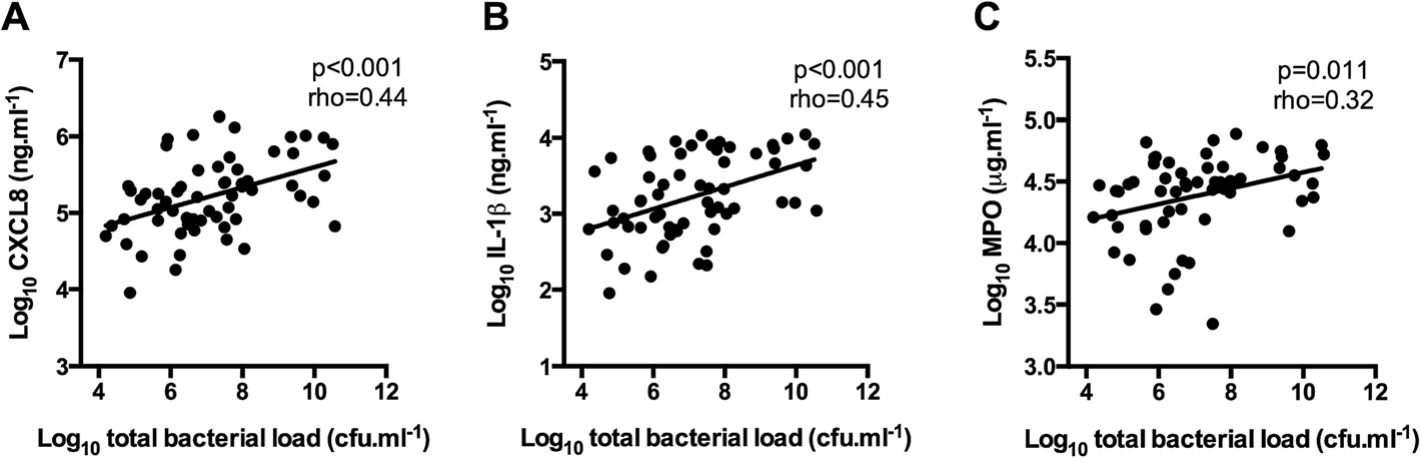
**Relationship between total bacterial load, as measured by qPCR, in colonised samples (n = 64) and (A) CXCL8, (B) IL-1β, and (C) MPO.**

Increasing total bacterial load was significantly associated with increasing CXCL8, IL-1β and MPO (rho = 0.44; p < 0.001, rho = 0.45; p < 0.001, and rho = 0.32; p = 0.011 respectively, Figure 2). All airway cytokines significantly correlated with each other (rho > 0.60; p < 0.001). There were no significant associations between airway cytokines and clinical demographics including FEV_1_ %predicted, exacerbation frequency, smoking status or pack year history (PYH), or inhaled corticosteroid use and dose (all p > 0.05, Additional file 1: Table S1).

**Figure 3:**
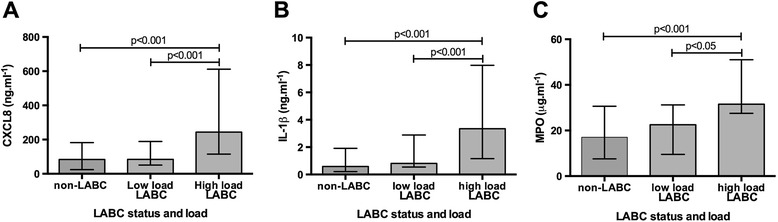
**Inflammatory thresholds of (A) CXCL8, (B) IL-1β, and (C) MPO.** Low load samples were defined as a total bacterial load of ≤10^7.0^ cfu.ml^-1^ (n = 32) and high load as >10^7.0^ cfu.ml^-1^ (n = 32). LABC = lower airway bacterial colonisation.

Using the previously proposed bacterial load-inflammatory threshold of 10^7^ cfu.ml^-1^ [10], LABC samples were sub-grouped into low total bacterial loads (≤10^7.0^ cfu.ml^-1^, n = 32) and high total bacterial loads (>10^7.0^ cfu.ml^-1^, n = 32) and compared with non-LABC samples. There was a significant difference between the three groups for all airway cytokines (p < 0.001). When each group was compared with each other, airway cytokines levels were similar between the non-LABC and low total bacterial load groups, but the high total bacterial load had significantly higher airway cytokines levels than both of these groups (Figure 3), suggesting a possible bacterial load threshold for increased airway inflammation.

Plasma fibrinogen was significantly higher in LABC samples than in non-LABC samples (3.8 (3.3-4.4) vs. 3.5 (3.2-4.1) g.l^-1^, p = 0.049, Table 2). There was no significant difference in serum CRP between non-LABC and LABC samples (2.0 (1.0-5.0) vs. 2.0 (1.0-8.0) mg.l^-l^, p = 0.261, Table 2).

### Species-specific inflammatory responses

**Figure 4:**
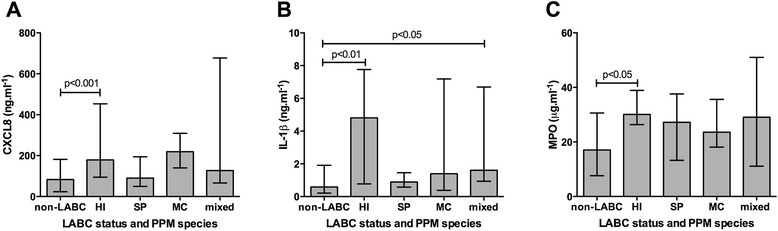
**The species-specific effect of potentially pathogenic microorganisms (PPMs) on (A) CXCL8, (B) IL-1β, and (C) MPO.** LABC = lower airway bacterial colonisation; HI = *Haemophilus influenzae*; SP = *Streptococcus pneumoniae*; MC = *Moraxella catarrhalis*; mixed = mixed potentially pathogenic microorganisms.

There was a significant difference in airway CXCL8, IL-1β and MPO between the samples with single isolation of of HI (n = 21), SP (n = 21) or MC (n = 7) and mixed-PPM (n = 15) colonised samples (p = 0.003, p < 0.001, and p = 0.011 respectively, Table 3). Despite similar bacterial loads in HI- and SP-colonised samples, and higher bacterial loads in MC- and mixed-PPM-colonised samples, CXCL8 was significantly higher only in HI-colonised samples compared to non-LABC samples (179 (95-453) vs. 83 (24-182) ng.ml^-1^, p < 0.001, Figure 4A). Furthermore, IL-1β and MPO levels were significantly higher in HI-colonised samples compared to non-LABC samples (4.8 (0.8-7.8) vs. 0.6 (0.2-1.9) ng.ml^-1^, p < 0.001 Figure 4B; 30.1 (26.4-38.9) vs. 17.1 (7.6-30.6) μg.ml^-1^, p < 0.05 Figure 4C).

**Table 3:** **Bacterial loads and associated airway inflammation in single and mixed-potentially pathogenic microorganisms (PPMs) colonised samples**

	**HI**	**SP**	**MC**	**mixed-PPM**	**p-value†**
	**n = 21**	**n = 21**	**n = 7**	**n = 15**	
**Mean (SD) Bacterial load**	10^6.2(1.2)^	10^6.5(1.1)^	10^8.6(1.8)^	10^8.5(1.5)^	<0.001
**Median (IQR) CXCL8 (ng.ml** ^**-1**^ **)**	179	90	219	127	0.003
	(95-453)	(50-194)	(140-309)	(67-677)	
**Median (IQR) IL-1β (ng.ml** ^**-1**^ **)**	4.8	0.9	1.4	1.6	<0.001
	(0.8-7.8)	(0.6-1.5)	(0.4-7.2)	(0.9-6.7)	
**Median (IQR) MPO (μg.ml** ^**-1**^ **)**	30.1	27.2	23.6	29.1	0.011
	(26.4-38.9)	(13.3-37.6)	(18.1-35.6)	(11.1-51.0)	

Definitions: HI = *Haemophilus influenzae*; SP = *Streptococcus pneumoniae*; MC = *Moraxella catarrhalis*; PPM = potentially pathogenic microorganisms.

^**†**^p-value refers to One-way ANOVA or Kruskal-Wallis between the single PPMs and mixed-PPM samples.

Mixed-PPM samples also had significantly higher IL-1β level than non-LABC samples (1.6 (0.9-6.7) vs. 0.6 (0.2-1.9) ng.ml^-1^, p < 0.05, Figure 4B). However, despite a higher total bacterial load in mixed-PPM colonised samples compared to HI-colonised samples, no significant augmentation of airway inflammatory responses was seen.

**Figure 5:**
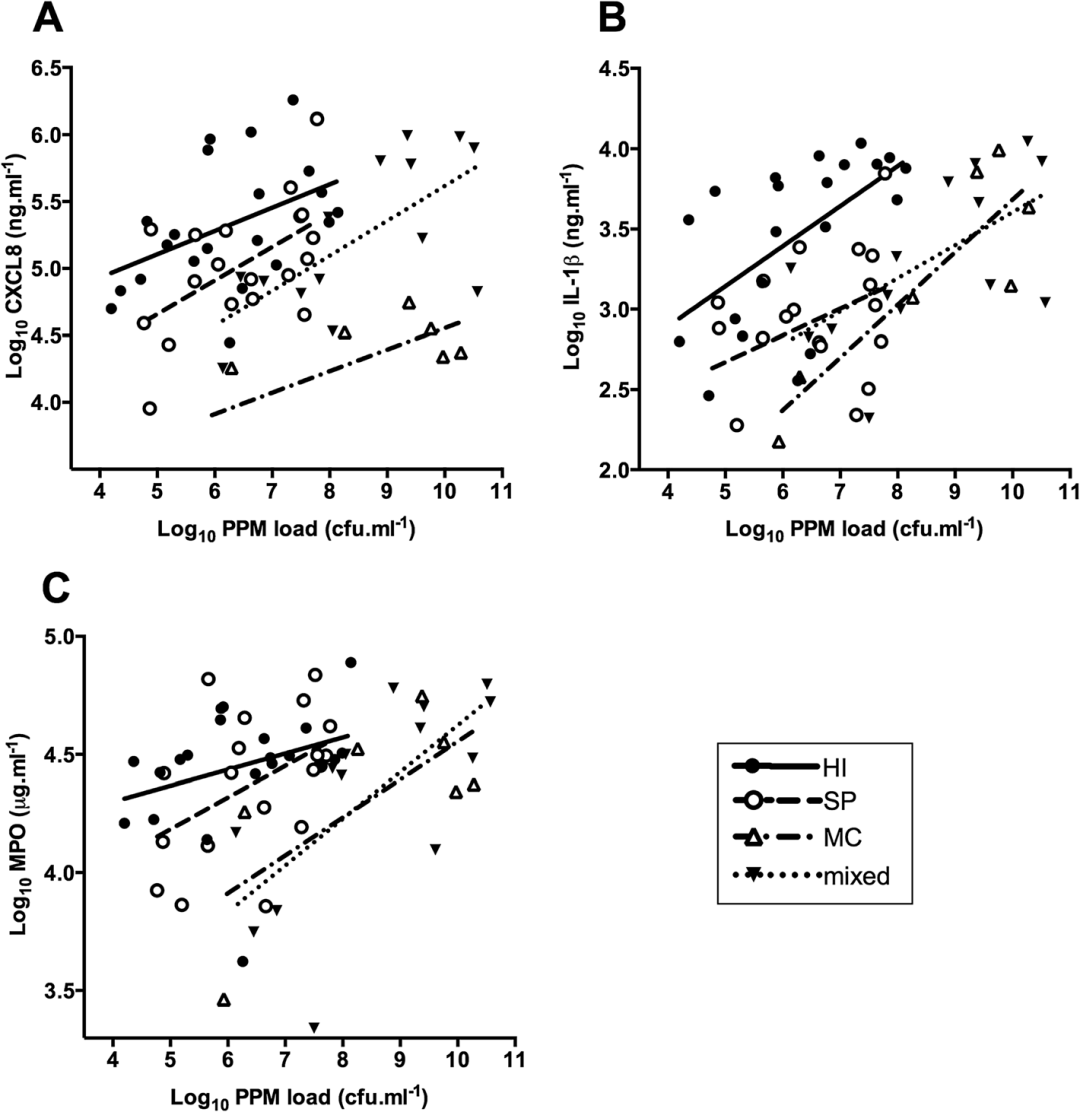
**Multiple regression analysis showing change in (A) CXCL8, (B) IL-1β, and (C) MPO in relation to the bacterial load of single isolate** ***Haemophilus influenzae*** **(HI, n = 21),** ***Streptococcus pneumoniae*** **(SP, n = 21) and** ***Moraxella catarrhalis*** **(MC, n = 7) and mixed-potentially pathogenic microorganisms (PPMs) (mixed, n = 15) samples.**

Adjusting for the different PPMs loads, HI-colonised samples were associated with significantly higher CXCL8, IL-1β and MPO response than individual SP or MC-colonised and mixed-PPM-colonised samples (p < 0.001, p < 0.001, and p = 0.002 respectively, Figure 5).

There were no species-specific differences in systemic inflammation as measured by serum CRP (p = 0.879) or plasma fibrinogen (p = 0.587).

### Bacterial colonisation and health status

74 SGRQs and 113 CAT scores were available with paired sputum samples. No significant association was observed between SGRQ or CAT scores with either airway or systemic inflammation in a univariate analysis or in a multivariate analysis taking into account FEV_1_ % predicted and exacerbation frequency (all, p > 0.05). There was no significant difference between total SGRQ and CAT scores in non-LABC and LABC samples (p > 0.05).

## Discussion

This is the first study to date to examine and validate the relationship between inflammation and airway PPM presence, load and species-effects using the culture-independent qPCR technique in a well-characterised cohort of stable COPD patients. We have demonstrated that the presence of sputum PPMs are associated with increased airway and systemic, as measured by fibrinogen, inflammatory response compared to samples without PPM detection. Increasing total bacterial load was associated with higher levels of airway but not systemic inflammation and importantly, we report an apparent inflammatory threshold. In addition, we have demonstrated a species-specific inflammatory response, with HI presence associated with significantly higher airway inflammatory response for all pathogen loads. However, there was discordance between health status and the airway inflammatory response.

Increasing qPCR total bacterial load was strongly associated with an increase in airway but not systemic inflammation. This was reflected by darker sputum colour which acts as a surrogate for MPO levels [21]. Importantly we have demonstrated an apparent total bacterial load threshold, above which airway inflammation is significantly higher than in samples without PPMs detected, or in those with lower loads, consistent with previous culture-based studies [10]. Detailed microbiomic studies in healthy, non-smoking individuals have demonstrated that a core pulmonary bacterial community exists which may include PPMs at low loads [15], and hence the detection of PPMs alone may not be pathological.

There is a long-standing debate as to the meaning of the term ‘colonisation’ [22]. This terminology suggests a benign process and one without host-pathogen interactions and consequences, which are key to the definition of infection. The importance of using a bacterial load threshold which is associated with a greater inflammatory response is that this avoids using purely the detection of a PPM to define colonisation, and thus enabling a more accurate definition which reflects the clear inflammatory consequences at higher bacterial loads. In this respect, it may be more appropriate to use the term ‘colonisation’ when PPMs are present, but in low loads and without inflammatory consequence, and ‘chronic airway infection’ when there are higher pathogen loads associated with a greater inflammatory response. We defined the threshold-effect using previous inflammatory data based on bacterial loads determined by culture [10]. However to date, there have been no direct comparisons of bacterial loads as measured by both culture and culture-independent microbiological techniques, and hence the bacterial load threshold measured by qPCR was an arbitrary measure. In our study, the colonised sample size may be underpowered to study this phenomenon in detail, and in particular with respect to the different PPMs. Therefore, further research is needed to investigate the presence of such a bacterial load threshold, using larger data sets, and different microbiological techniques, and to determine how different bacterial loads may affect this inflammatory threshold.

Systemic inflammation, as measured by fibrinogen but not CRP, was significantly higher in samples with PPMs detected than in those without, although the absolute difference was small. No relationship was observed between either total bacterial load or species-specific loads and systemic inflammation. The majority of previous studies investigating inflammatory response in stable COPD patients have focused on airway rather than systemic inflammation [6,10,11,13]. When systemic inflammation has been measured in patients with PPM detection, the evidence for raised fibrinogen and CRP has been conflicting, and no clear association has been found with bacterial loads [7,12]. However, systemic inflammation, and in particular raised fibrinogen, has been suggested as a possible link between COPD and associated cardiovascular events [23] and also as a potential biomarker to identify patients with a higher risk of mortality [24]. Therefore, the role of fibrinogen in the pathogenesis and clinical outcomes in COPD patients with bacterial colonisation is important area of research for future studies.

We demonstrated greater airway inflammation for all measured cytokines appears to be attributable to HI rather than other PPMs. Species-specific effects on airway inflammation have been alluded to in an *in vitro* study of MC [25], and also with HI in culture-based *in vivo* studies of patients with chronic bronchitis [10] and in stable COPD patients with HI colonisation [12]. However, these studies did not take into account the different pathogen loads. We have demonstrated that individual pathogens and mixed-PPM samples had significantly different loads, and at all bacterial loads, airway CXCL8, IL-1β, and MPO were significantly higher for HI.

HI is often the most prevalent PPM cultured at stable state in COPD patients [6,11-13], and a key mechanism in its pathogenesis is its ability to adhere to the already damaged epithelium of the lower respiratory tract in COPD patients [26], and some studies have demonstrated that HI may reside between the epithelial and subepithelial tissues [27], evading mucosal immunity and worsening the underlying airway inflammation seen in COPD.

There was evidence in this study of discordance between inflammation and health status as measured by the SGRQ and CAT scores, and therefore targeting inflammation alone may not necessarily improve health status. However, a recent longitudinal study by Desai and colleagues demonstrated that daily symptoms, as measured by the Breathlessness, Cough and Sputum Scale (BCSS), were significantly higher during periods of colonisation compared to periods without [28]. PPM isolation in stable COPD has been shown to be associated with an increased exacerbation frequency [6] and faster decline in FEV_1_ % predicted [13], both of which contribute significantly to health status [17]. Therefore, the lack of correlation between quality of life and colonisation in our study compared to the study by Desai and colleagues may be explained by the longitudinal nature of their study and different heath status questionnaires used. Further longitudinal studies are warranted to determine whether these important clinical outcomes are also species-specific, and thus whether targeting stable HI isolation by using long-term antibiotics or vaccination would provide clinical benefit.

PPM detection during stable COPD appears to be dynamic, with patients changing their colonisation status and which species, strain and load of PPMs are isolated, likely to result in waxing and waning of airway inflammation [13,29-31]. This study was limited to cross-sectional analysis, with lower airway bacterial colonisation defined as being a single positive sputum sample. The inflammatory responses to bacterial loads as measured by molecular techniques have not been previously reported, but in view of the high diagnostic yield of this qPCR technique compared to culture, further studies exploring this dynamic process are likely to use similar techniques, and therefore the relationship between load and inflammation must first be explored. Unlike in other respiratory diseases, such as cystic fibrosis, where multiple positive sputum samples are required before patients are classified as ‘colonised’, there has been no such consensus in COPD research studies. Thus, further longitudinal studies must aim to address this key question as to whether single or multiple positive sputum samples define colonisation, so appropriate patients can be targeted for clinical trials.

Although qPCR may detect both viable and non-viable bacteria, a clear relationship between bacterial DNA load and airway inflammation is demonstrated. While this qPCR technique does not detect PPMs other than HI, SP, or MC, the corresponding routine culture data showed that only 2% of sputum samples had evidence of a PPM other than those that are able to be detected by this technique. However, routine bacterial culture only reports PPMs with a bacterial load greater than 10^5^ cfu/ml, and therefore some patients who were determined to be non-colonised, may indeed have PPMs present at stable state, contributing to the total bacterial load. Although total bacterial loads, as measured by quantitative bacterial culture and culture-independent techniques, such as 16S, are able to detect both recognised respiratory pathogens and commensal bacteria, these techniques also have recognised limitations. Quantitative culture has the inherent inaccuracy of visual interpretation of colony counts, and the interaction between non-respiratory pathogens, included in the total bacterial load as measured by 16S, and inflammation is not well characterised. However, despite these limitations, the high diagnostic yield of this qPCR technique highlights the strength of this technique and its potential utility in the clinical practice of microbiological study.

A further limitation in our study is that co-existing bronchiectasis was not assessed by CT scanning, although no patients had evidence of clinical bronchiectasis. Previous studies report up to 50-60% of stable COPD have radiological evidence of bronchiectasis, although these changes were generally mild [32,33]. Secondary bronchiectasis may be more likely in COPD patients with airway PPM isolation as a result of the vicious cycle of inflammation and infection characteristic of LABC, and thus the availability of CT scans would be unlikely to alter the important findings from this study.

In conclusion, using the high diagnostic yield of qPCR to identify airway PPMs and their loads, we have demonstrated that the airway, but not systemic inflammatory response, appears to have an inflammatory threshold and is significantly higher with HI presence. Further observational and interventional evidence is required to understand the nature and significance of dynamic changes in HI and other PPMs.

## Additional file

Additional file 1: Table S1. Relationships between airway inflammation and clinical demographics from 99 stable COPD patients*.

## Abbreviations

ATS: American thoracic society
BMI: Body mass index
CAT: COPD assessment test
cfu: colony-forming units
COPD: Chronic obstructive pulmonary disease
CRP: C-reactive protein
CXCL8: CXC-chemokine ligand 8
ERS: European respiratory society
FEV_1_: Forced expiratory volume in one second
FVC: Forced vital capacity
HI: *Haemophilus influenzae*
ICS: Inhaled corticosteroids
IL: Interleukin
LABC: Lower airway bacterial colonisation
MC: *Moraxella catarrhalis*
MPO: Myeloperoxidase
PPMs: Potentially pathogenic microorganisms
PYH: Pack year history
qPCR: Quantitative polymerase chain reaction
SGRQ: St George’s respiratory questionnaire
SP: *Streptococcus pneumoniae*
