# Supplementary material for: Inflammatory thresholds and the species-specific effects of colonising bacteria in stable chronic obstructive pulmonary disease
Source: Respir Res. 2014 Sep 14;15(1):114. doi: 10.1186/s12931-014-0114-1 (PMC4173051; doi:10.1186/s12931-014-0114-1)
Supplement: Additional file 1: Table S1. — Relationships between airway inflammation and clinical demographics from 99 stable COPD patients*. [file 12931_2014_114_MOESM1_ESM.docx]

**Additional file 1: Table S1**. Relationships between airway inflammation and clinical demographics from 99 stable COPD patients*.

*****Only the first sample was used from each patient to avoid complications with repeated measures.

Definitions: ICS = inhaled corticosteroids.

| **Clinical demographic** |  | **p-value** |  |
| --- | --- | --- | --- |
|  | **CXCL8** | **IL-1β** | **MPO** |
| **FEV1 %predicted†** | 0.917 | 0.882 | 0.137 |
|  |  |  |  |
| **Exacerbation frequency†** | 0.172 | 0.211 | 0.658 |
|  |  |  |  |
| **Pack year history†** | 0.182 | 0.843 | 0.085 |
|  |  |  |  |
| **Smoking status§** | 0.205 | 0.337 | 0.267 |
| **ICS use**** | 0.543 | 0.217 | 0.756 |
| **ICS dose†** | 0.727 | 0.538 | 0.484 |

**†**p-value refers to Spearman’s rank correlation between airway cytokines and clinical demographics.

§p-value refers to Mann-Whitney U test between current smokers and ex-smokers.

** p-value refers to Mann-Whitney U test between patients with concurrent ICS use and those without concurrent ICS use.
